# Cullin 5 is a novel candidate tumor suppressor in renal cell carcinoma involved in the maintenance of genome stability

**DOI:** 10.1038/s41389-018-0110-2

**Published:** 2019-01-09

**Authors:** María Ángeles Tapia-Laliena, Nina Korzeniewski, Samuel Peña-Llopis, Claudia Scholl, Stefan Fröhling, Markus Hohenfellner, Anette Duensing, Stefan Duensing

**Affiliations:** 1 Section of Molecular Urooncology, Department of Urology, University of Heidelberg School of Medicine, Im Neuenheimer Feld 517, D-69120 Heidelberg, Germany; 2 Department of Translational Medical Oncology, National Center for Tumor Diseases (NCT) Heidelberg, German Cancer Research Center (DKFZ), Im Neuenheimer Feld 460, D-69120 Heidelberg, Germany; 3 Division of Applied Functional Genomics, German Cancer Research Center (DKFZ), Im Neuenheimer Feld 581, D-69120 Heidelberg, Germany; 4 Department of Urology, University of Heidelberg School of Medicine, Im Neuenheimer Feld 110, D-69120 Heidelberg, Germany; 5 Cancer Therapeutics Program, UPMC Hillman Cancer Center and Department of Pathology, University of Pittsburgh School of Medicine, UPMC Hillman Cancer Center, 5117 Centre Avenue, Pittsburgh, PA 15213 USA; 6 Present Address: Division of Solid Tumor Translational Oncology, West German Cancer Center (WTZ), Essen University Hospital, German Cancer Consortium (DKTK) at Essen/Düsseldorf, Hufelandstrasse 55, D-45147 Essen, Germany

## Abstract

Clear cell renal cell carcinoma (ccRCC) is intimately associated with defects in ubiquitin-mediated protein degradation. Herein, we report that deficiency in the E3 ligase subunit cullin 5 (CUL5) promotes chromosomal instability and is an independent negative prognostic factor in ccRCC. CUL5 was initially identified in an RNA interference screen as a novel regulator of centrosome duplication control. We found that depletion of CUL5 rapidly promotes centriole overduplication and mitotic errors. Downregulation of CUL5 also caused an increase of DNA damage that was found to involve impaired DNA double-strand break repair. Using immunohistochemistry, CUL5 protein expression was found to be below detection level in the majority of RCCs. A re-analysis of the TCGA ccRCC cohort showed that a reduced CUL5 gene expression or *CUL5* deletion were associated with a significantly worse overall patient survival. In conclusion, our results indicate that CUL5 functions as a novel tumor suppressor with prognostic relevance in ccRCC and is critically involved in the maintenance of genome stability.

## Introduction

Kidney cancer affects over 300,000 people worldwide annually and is one of the most lethal urological malignancies once metastatic^1^. Clear cell renal cell carcinoma (ccRCC) is the most common histological subtype and is thought to arise from cells lining the proximal tubule of the nephron^2^.

Like most solid tumors, ccRCC is characterized by chromosomal instability including numerical and structural chromosomal alterations^3^. Some of these alterations such as the loss of chromosome 3p are highly characteristic for ccRCC^4,5^. While loss of chromosome 3p has been suggested to represent an early event in ccRCC^4^, there is an association between chromosomal complexity and metastatic disease as highlighted by the frequent coincidence of loss of chromosomes 9p and 14q in advanced stage disease^6^. Whole chromosome copy number changes (aneuploidy) are also frequent findings in ccRCC, which, together with structural changes and single-nucleotide variants^7^ contribute to the extensive intratumoral genetic heterogeneity characteristic of ccRCC^8,9^.

In general, numerical and structural chromosomal aberrations are caused by mitotic defects and errors in DNA damage repair, respectively, which frequently coincide in cancer cells^10^.

In ccRCC, the inactivation of the *VHL* tumor suppressor gene, which occurs in the large majority of patients, has been shown to lead to defective mitoses and also to interfere with DNA double-strand break (DSB) repair^11,12^. The pVHL protein is part of a protein complex that includes elongin B, elongin C, Rbx1 and cullin 2 and functions as E3 ubiquitin ligase^13–15^. Cullin RING E3 ubiquitin ligases (CRLs) constitute the major subfamily of E3 ligases and play an important role in the ubiquitin-mediated protein turnover in cells. CRLs are characterized by a common cullin-containing scaffold protein^15^. There are eight human cullin subunits (CUL1, -2, -3, -4A, -4B, -5, -7 and PARC) which orchestrate the assembly of unique ubiquitin ligase complexes. All CRLs consist of a cullin-backbone, a zinc-binding RING-domain containing protein, which recruits the ubiquitin-conjugating E2 enzyme, and an adaptor protein that binds interchangeable substrate recognition subunits, which provide target specificity to each individual CRL^15–17^.

Another main tumor suppressor gene in ccRCC is the deubiquitinase BAP1, which is inactivated in about 15% of patients^18^ and, among other functions, promotes DNA DSB repair^19^. Whether and to what extent the loss of additional tumor suppressors involved in ubiquitin-proteasome-mediated protein degradation contribute to chromosomal instability in ccRCC is a matter of ongoing research^20^.

Herein, we show that CUL5 is a novel candidate tumor suppressor in ccRCC. Our results show that CUL5 is critically involved in the regulation of centriole duplication and DNA damage repair, and that loss of expression is a negative prognostic factor in ccRCC patients. Our findings highlight the central role of CRLs, including CUL5, in RCC development and progression.

## Results

### Downregulation of CUL5 promotes centriole overduplication

**Fig. 1:**
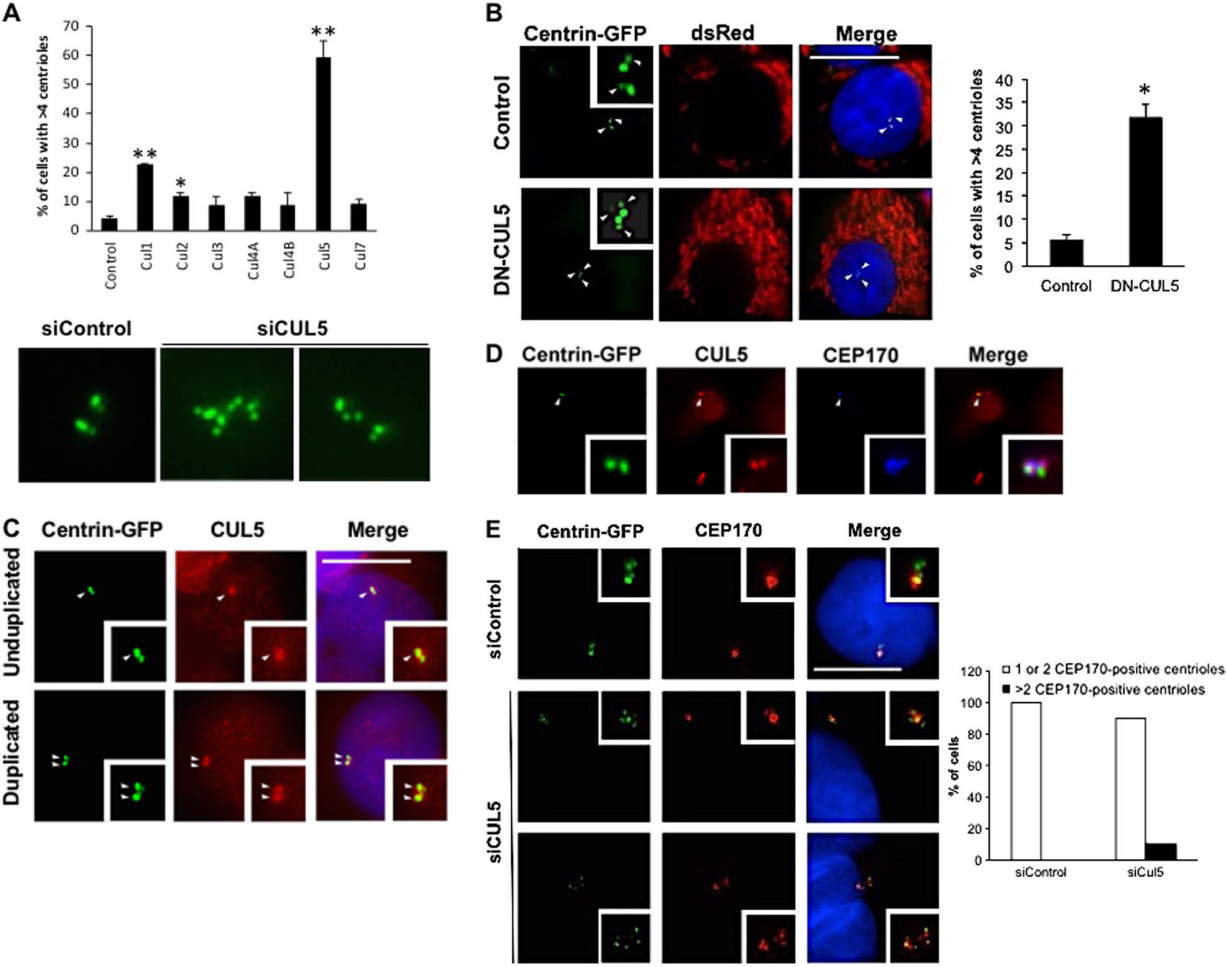
CUL5 restricts centriole duplication. **a** (upper panel) Quantification of cells with >4 centrioles following siRNA-mediated depletion of human cullin subunits in U-2 OS/centrin-GFP cells (72 h). Each bar represents mean plus standard error of at least two independent experiments with a minimum of 300 cells counted per experiment. Asterisks indicate statistically significant differences (*p ≤ 0.05; **p ≤ 0.005). **a** (lower panel) Fluorescence microscopic analysis of U-2OS cells stably expressing centrin-GFP and transiently transfected with control siRNA or siRNA duplexes targeting CUL5. Note the presence of supernumerary daughter centrioles after CUL5 knock-down as indicated by the weaker GFP signals. **b** Fluorescence microscopic analysis (left panels) and quantification (right panel) of U-2 OS/centrin-GFP cells transfected with either DN-CUL5 or a control plasmid (48 h). dsRed was used as transfection marker. Arrows indicate daughter centrioles. Scale bar = 10 μm. Mean and standard error of three independent experiments with at least 100 cells counted per experiment are shown. **c** Immunofluorescence microscopic analysis of U-2 OS/centrin-GFP cells for CUL5. Note the association of CUL5 with the bigger one of the two unduplicated centrioles (arrow, top panels) and with the second centriole in cells with duplicated centrioles (arrows, bottom panels). Scale bar = 10 μm. **d** Co-immunofluorescence microscopic analysis of U-2 OS/centrin-GFP cells for CUL5 and CEP170. Note the enhanced co-localization of CUL5 with CEP170 at the older, mature centriole. Arrowheads point to the areas shown in insets. **e** Immunofluorescence microscopic analysis (left panels) and quantification (right panel) of U-2 OS centrin/GFP cells with centriole overduplication in the presence of 1 or 2 or >2 CEP170-positive centrioles following siRNA-mediated depletion of CUL5 (72 h). Scale bar = 10 μm

To explore the role of cullins in the maintenance of mitotic fidelity, we performed a small interfering RNA (siRNA) mini-screen of seven human cullin subunits. Protein knock-down was performed in U-2 OS cells stably expressing centrin-green fluorescent protein (U-2 OS/centrin-GFP; Fig. 1a; Suppl. Figure 1). This allows the visualization of centrioles, the core forming units of centrosomes, which serve as the major microtubule-organizing centers in most mammalian cells in interphase and mitosis. We found that knock-down of CUL5 leads to an overduplication of centrioles in a very high percentage of cells (56.9%, *p* ≤ 0.001; Fig. 1a). This increase is among the highest reported so far after transient manipulation of cells and only comparable to PLK4 (polo-like kinase 4) overexpression, one of the strongest stimuli for centriole overduplication known so far^21,22^. Following depletion of CUL5, we observed several centriole overduplication defects including centriole multiplication and daughter–daughter pairs (Fig. 1a, bottom panels).

We also used a dominant-negative truncation mutant of CUL5 (DN-CUL5), which effectively reduces E3 ligase activity^23^, to transiently transfect U-2 OS/centrin-GFP cells. An increase in centriole overduplication from 6.6% in controls to 29% in DN-CUL5 transfected cells (*p* *≤* 0.001; Fig. 1b) was detected.

In order to prove that CUL5-based E3 ubiquitin ligase activity also restrained centriole biogenesis in non-transformed cells, we generated a CUL5 short-hairpin RNA (shRNA)-expressing stable cell lines using normal BJ fibroblasts expressing the catalytic subunit of telomerase (BJ/TERT) and analyzed centriole numbers. Knock-down of CUL5 by shRNA increased the number of cells with centriole overduplication to 21.5% as compared to 4% with the shRNA control vector (*p* ≤ 0.001; Suppl. Figure 2). This confirms our previous results with siRNA experiments in U-2 OS centrin-GFP cells.

We next analyzed the cellular localization of CUL5 in U-2 OS/centrin-GFP cells by fluorescence microscopy. We found CUL5 to localize to centrioles (Fig. 1c). This co-localization pattern between centrin-GFP and CUL5 suggested that CUL5 may be present mostly at mature centrioles. Co-staining of CUL5 and CEP170, a marker for older, mature centrioles^24^, in U-2 OS/centrin-GFP cells confirmed that CUL5 does show a more predominant expression at older, mature centrioles (Fig. 1d).

### Knock-down of CUL5 produces a genuine centriole duplication defect

When we performed an immunofluorescence microscopic analysis of U-2 OS/centrin-GFP cells for γ-tubulin after depletion of CUL5 by siRNA, we found a sixfold increase in the number of γ-tubulin dots per cell (not shown) underscoring that supernumerary centrioles induced by knock-down of CUL5 undergo maturation and can hence potentially function as microtubule-organizing centers in cells.

Genuine centriole overduplication is characterized by the presence of one or two mature centrioles and an abnormal number of immature daughter centrioles^21,24^. In contrast, centriole accumulation is characterized by the presence of multiple maternal centrioles with a normal mother–daughter centriole ratio^21,24^. We found that only a small fraction of cells (10%; Fig. 1e) in asynchronously growing, CUL5-depleted U-2 OS/centrin-GFP cells contained an abnormal number of CEP170-positive centrioles (>2 CEP170-positive centrioles) in the presence of an increased number of immature (CEP170-negative) centrioles (Fig. 1e), demonstrating that aborted mitosis or cytokinesis errors are not a major mechanism behind the centriole overduplication in cells depleted of CUL5.

Taken together, these results suggest that CUL5 depletion results in an increase in supernumerary centrioles through genuine disruption of the centriole duplication cycle and that a significant fraction of these overduplicated centrioles are capable of recruiting γ-tubulin, indicating that they are functional.

### CUL5 is necessary to maintain mitotic fidelity

**Fig. 2:**
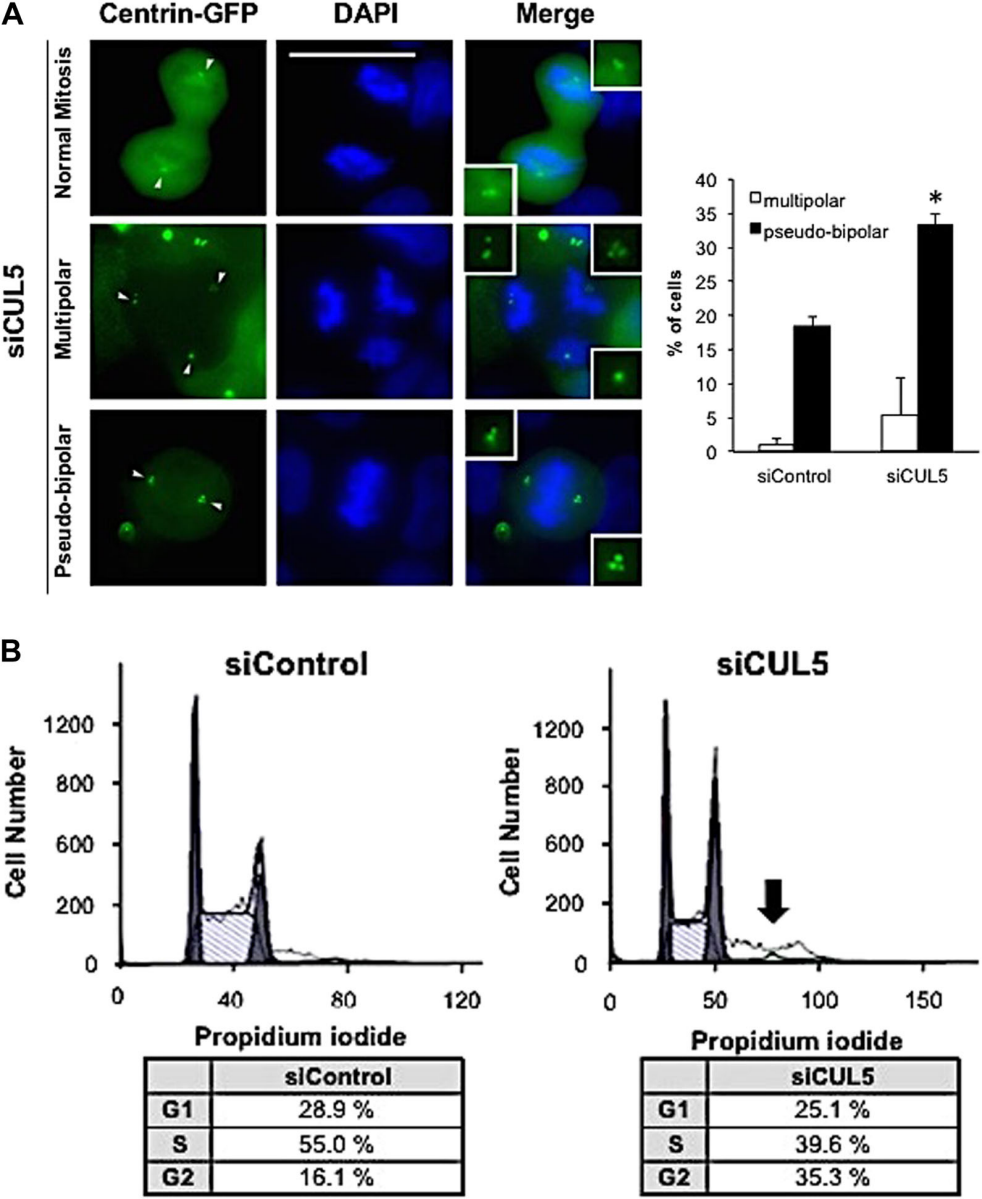
CUL5 is required to maintain mitotic fidelity. **a** Fluorescence microscopic analysis (left panel) and quantification (right panel) of abnormal mitotic cells after siRNA-mediated depletion of CUL5 in U-2OS/centrin-GFP cells (96 h). Insets show the spindle poles. Scale bar = 10 μm. Each bar represents mean plus standard error of at least two independent experiments with a minimum of 100 cells counted per experiment. **b** Flow cytometric analysis of U-2 OS/centrin-GFP cells following siRNA-mediated depletion of CUL5 (siCUL5) or control siRNA (siControl) for 72 h. Arrow indicates poly-/aneuploid cells. Asterisks indicate statistically significant differences (*p ≤ 0.05)

We next determined the consequences of CUL5 knock-down on mitotic fidelity. CUL5 depletion significantly increased the percentage of cells exhibiting abnormal mitosis (multipolar and pseudo-bipolar combined) from 19% in control cells to 38% in CUL5 siRNA-transfected cells (*p* *≤* 0.001). Whereas multipolar mitoses increased from 1% in controls to 5% in CUL5-depleted cells, the proportion of pseudo-bipolar mitoses increased from 18% in controls to 33% in CUL5-depleted cells (*p* ≤ 0.05; Fig. 2a).

These results further underscore that supernumerary centrioles induced by CUL5 depletion are functional and can promote abnormal mitoses, thereby potentially promoting chromosomal instability in daughter cells.

Flow cytometric analysis of the DNA content of CUL5-depleted cells showed an increase in the number of cells in the G_2_/M phase of the cell division cycle (35.3%) compared to control siRNA-treated cells (16.1%) (*p* ≤ 0.001; Fig. 2b). There was also an increase of cells with >4N DNA content, indicating the presence of polyploid/aneuploid tumor cells following CUL5 depletion.

**Fig. 3:**
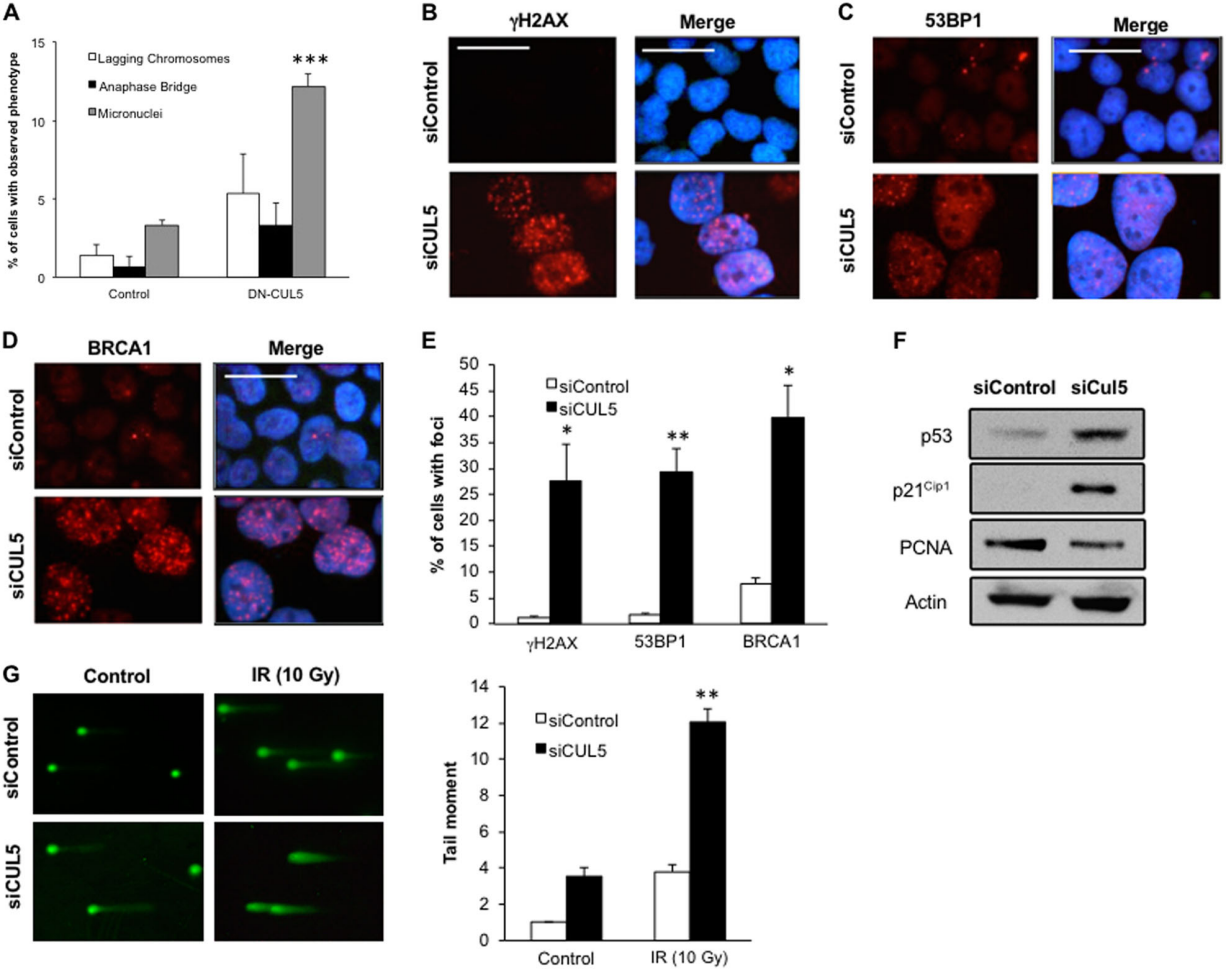
CUL5 is required for the maintenance of structural chromosomal integrity. **a** Quantification of morphological cellular changes suggestive of DNA damage in U-2 OS/centrin-GFP cells after transfection with either control plasmid or DN-CUL5. Note the significant increase of micronuclei in cells with impaired CUL5 function. Each bar indicates mean and standard error of two independent experiments with at least 100 cells counted per experiment. **b–d** Immunofluorescence microscopic analysis of U-2 OS/centrin-GFP cells for markers of DNA damage (γH2AX, 53BP1 and BRCA1 nuclear foci) following siRNA-mediated depletion of CUL5 (72 h). Nuclei were stained with DAPI. Scale bars indicate 10 μm. **e** Quantification of cells with nuclear foci of the indicated DNA damage markers following siRNA-mediated depletion of CUL5 in U-2 OS/centrin-GFP cells (72 h). Each bar represents mean plus standard error of at least two independent experiments with a minimum of 300 cells counted per experiment. **f** Immunoblot analysis of U-2 OS/centrin-GFP cells for p53, p21^Cip1^, PCNA and actin as loading control after transfection of cells with control siRNA or siRNA targeting CUL5 (72 h). **g** Fluorescence microscopic analysis (left panels) and quantification (right panel) of DNA breakage in U-2 OS/centrin-GFP cells following transfection with control siRNA or siRNA-mediated depletion of CUL5 (72 h) and 1 h after exposure to 10 Gy ionizing irradiation (IR) using the Comet assay. Each bar represents mean plus standard error of at least two independent experiments with a minimum of 300 cells counted per experiment. Asterisks indicate statistically significant differences (*p ≤ 0.05; **p ≤ 0.005; ***p ≤ 0.0005)

Moreover, we detected signs of structural DNA aberrations in cells with abrogated CUL5 function, including lagging chromosomes, anaphase bridges and micronuclei (Fig. 3a). In particular, there was a significant increase of cells containing micronuclei from 3.3% in controls to 12.2% in cells transfected with DN-CUL5 (*p* ≤ 0.0001; Fig. 3a).

Taken together, our results show that depletion of CUL5 rapidly causes mitotic defects and chromosome segregation errors but also alterations suggestive of additional structural chromosomal damage.

### CUL5 is required for DNA damage repair

Having shown that CUL5 depletion leads to a significant increase of cells with micronuclei, we next explored a possible role of CUL5 in the cellular response to DNA damage.

First, we examined the formation of DNA damage-associated foci containing γH2AX (Fig. 3b), 53BP1 (Fig. 3c) or BRCA1 (Fig. 3d) after depletion of CUL5 by siRNA in U-2 OS/centrin-GFP cells. We detected a 22.9-fold increase of γH2AX foci (from 1.2% to 27.5%; Fig. 3e), a 16.2-fold increase of 53BP1 foci (from 1.8% to 29.2%; Fig. 3e), and a 5.1-fold increase of BRCA1 foci (from 7.9% to 39.9%; Fig. 3e). This DNA damage response was accompanied by a cellular stress response including an upregulation of p53 and its transcriptional target p21^Cip1^ as well as reduced cellular proliferation (Fig. 3f).

We next asked whether CUL5 depletion can directly induce DNA strand breaks or whether it rather interferes with the repair of DNA strand breaks. To this end, we performed a Comet assay to quantify the DNA damage in U-2 OS cells after CUL5 siRNA transfection and exposure to 10 Gy of ionizing radiation (IR; Fig. 3g). In particular, the Comet tail moment, which is defined by the product of the tail length and the fraction of total DNA in the tail, represents a suitable measure of the extent of DNA damage. We found an increase of the tail moment in CUL5 siRNA-transfected cells compared to controls in the absence of IR, suggesting that CUL5 depletion by itself can induce DNA breakage. However, a significant increase of the tail moment was detected after prior exposure to IR, suggesting that CUL5 may also interfere with the DNA strand break repair after exogenous DNA damage (Fig. 3g).

Taken together, these results show that CUL5 downregulation promotes DNA breakage and interferes with DNA break repair.

### Frequent loss of CUL5 protein expression in renal cell carcinoma

Since CUL5 shares the substrate recognition adaptor and the SOCS/BC box protein substrate receptor with CUL2, which has already been implied in renal carcinogenesis through von Hippel–Lindau (VHL)^25,26^, we decided to study the potential role of CUL5 in RCC progression in greater detail.

**Fig. 4:**
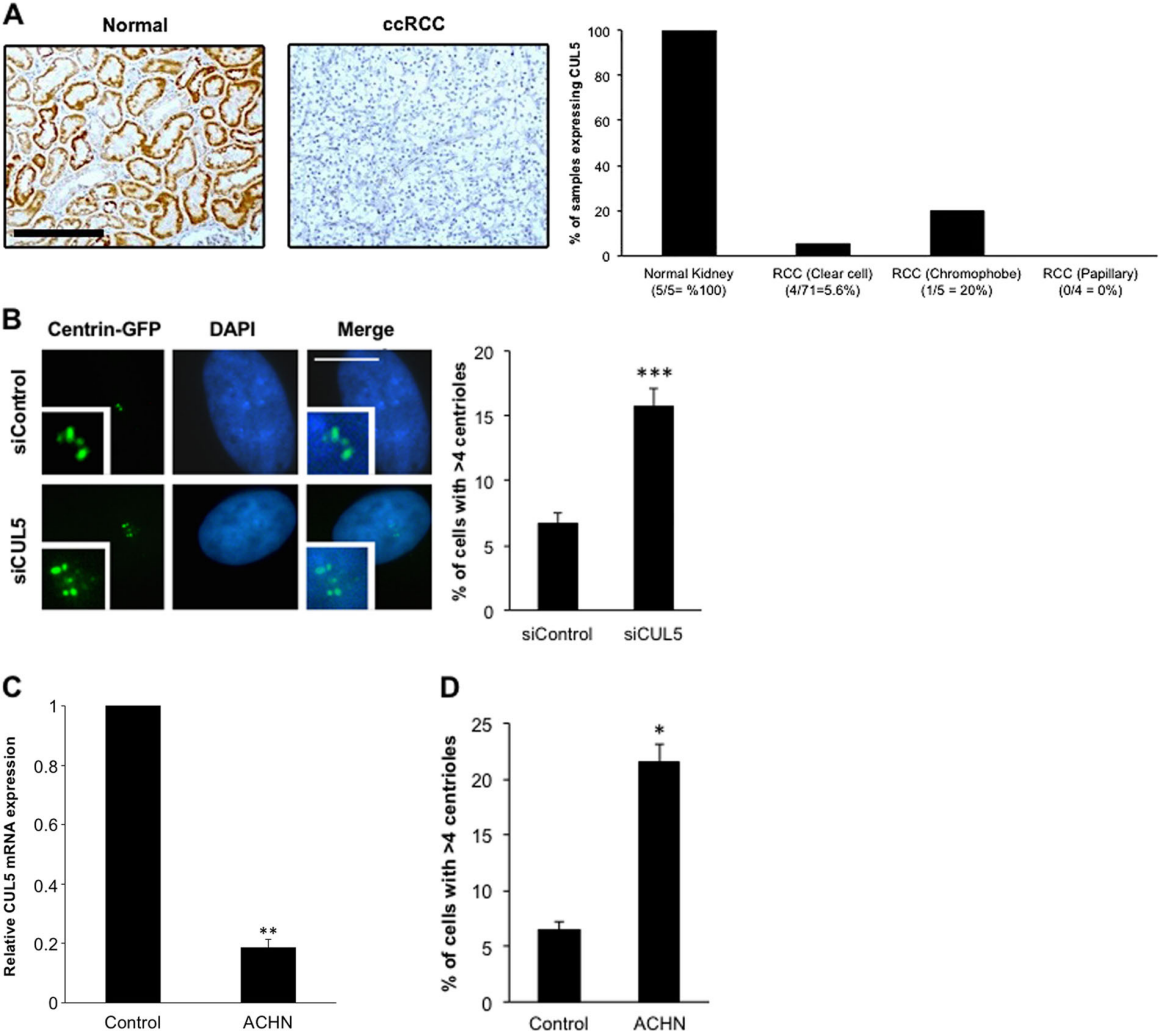
Loss of CUL5 expression in renal cancer. **a** Immunohistochemical staining (left panels) and quantification (right panel) of CUL5 expression using a renal cell carcinoma tissue microarray. Representative immunostainings of CUL5 in human normal kidney and a ccRCC are shown. Scale bar = 250 μm. **b** Fluorescence microscopic analysis (left panels) and quantification (right panel) of normal human renal epithelial cell (hRECs) for centriole numbers following siRNA-mediated depletion of CUL5 and co-transfection with a plasmid that expresses centrin-GFP (72 h). Each bar represents mean plus standard error of at least two independent experiments with a minimum of 300 cells counted per experiment. **c** Quantitative real-time reverse transcriptase polymerase chain reaction (qRT-PCR) for CUL5 mRNA expression in normal human kidney cells (HEK293) used as controls and ACHN cells. β-Actin was used as a control housekeeping gene. Five replicates were performed. **d** Quantification of cells with spontaneous centriole overduplication in primary human renal epithelial cells (Control) in comparison to ACHN cells. Centrioles were visualized by transfection with a plasmid encoding centrin-GFP (48 h). Mean and standard error of two independent experiments with at least 100 cells counted per experiment are shown. Asterisks indicate statistically significant differences (*p ≤ 0.05; **p ≤ 0.005; ***p ≤ 0.0005)

First we analyzed the protein expression of CUL5 in RCC tissue samples by immunohistochemistry (Fig. 4a). Whereas normal kidney tissue was consistently positive for CUL5, only 4 of 71 ccRCCs, 1 of 5 chromophobe RCCs and no papillary RCC showed expression of CUL5 (Fig. 4a, right panel).

We next suppressed CUL5 messenger RNA (mRNA) level in normal human renal epithelial cells (hRECs) using siRNA and found that depletion of CUL5 promoted centriole overduplication in 15.6% of cells compared to 6.6% of control cells (*p* ≤ 0.0001; Fig. 4b). Next, we examined CUL5 mRNA level in normal human kidney cells versus the metastatic renal carcinoma cell line ACHN, which shows mixed papillary and clear cell features^27^. ACHN cells have also been tested for mutations in *CUL5* and were found to be negative except for a silent mutation in exon 3^28^. ACHN cells showed a significantly reduced CUL5 mRNA expression than normal human kidney cells cells (*p* *≤* 0.005; Fig. 4c). In line with a reduced amount of CUL5 mRNA, the ACHN cell line also exhibited a high percentage of cells, i.e., 21.5% with centriole overduplication compared to 6.5% in normal human kidney cells (*p* ≤ 0.05; Fig. 4d).

Since the metastatic ACHN cells present low CUL5 mRNA levels together with a high percentage of centriole overduplication, compared to normal hRECs, CUL5 may be an important factor for the progression of renal carcinoma.

### Loss of CUL5 gene expression is associated with an unfavorable prognosis in RCC patients

**Fig. 5:**
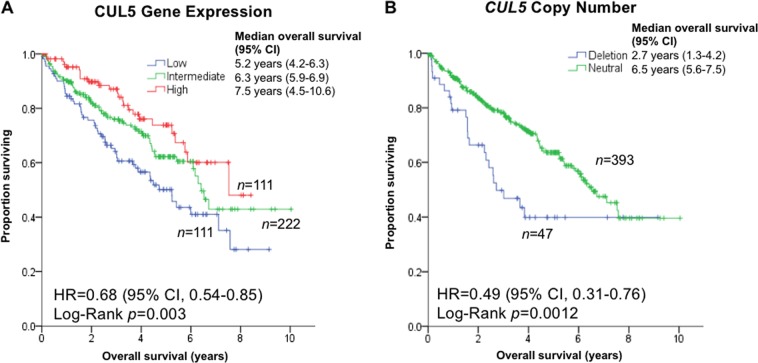
Low CUL5 expression is associated with impaired survival in patients with ccRCC. **a** Kaplan–Meier survival curve of KIRC-TCGA patients showing low CUL5 gene expression (1st quartile, blue), which is associated with significantly worse overall overall survival when compared to patients with high CUL5 expression levels (4th quartile, red). Second and third quartiles are shown combined in green. **b** Kaplan–Meier survival curve of KIRC-TCGA patients with a *CUL5* chromosomal deletion in comparison to patients with no loss of the *CUL5* gene. Note the significantly worse overall survival in patients with a *CUL5* deletion

To further corroborate the notion that a loss of CUL5 may play a role in the progression of ccRCC, we analyzed The Cancer Genome Atlas (TCGA) Kidney Renal Clear Cell Carcinoma (KIRC) cohort, a large patient cohort (*n* > 400) for which both clinical and genomic data are available (Fig. 5).

Patients harboring tumors with low expression levels of CUL5 displayed a shorter survival (a median of 5.2 years) compared to high CUL5 expressing tumors (a median of 7.5 years) (*p* = 0.003, Fig. 5a). Patients with chromosomal deletions in the *CUL5* locus showed a significantly impaired cancer-specific survival (median of 2.7 years compared to 6.5 years, *p* = 0.0012, Fig. 5b).

To investigate the potential of CUL5 levels as biomarker of prognosis, we performed univariate Cox regression models on the clinical variables and CUL5 gene expression levels (Table 1). Variables that were significant in the univariate Cox model were entered into an unsupervised stepwise forward conditional multivariate Cox analysis to identify independent predictors of survival. Advanced age (higher than median), high grade, metastasis at diagnosis (M+), high tumor stage and low CUL5 expression were independently associated with poor survival in ccRCC patients by being present in the final step of the multivariate Cox model. The same variables were retained in a stepwise backward conditional multivariate analysis.

**Table 1:** Univariate and multivariate Cox regressions of the clinical variables and CUL5 expression from the KIRC-TCGA dataset

	Univariate analysis			Multivariate analysis		
Variable	HR	95% CI	*P*	HR	95% CI	*P*
Age (>median)	2.0	1.4–2.7	8·10^−5^	2.0	1.3–3.3	0.004
Gender (male)	0.8	0.6–1.2	0.32			
High grade (3–4 vs. 1–2)	2.3	1.6–3.3	7·10^−6^	1.7	1.0–2.9	0.03
High tumor size (>median)	2.7	1.9–3.8	4·10^−8^			
High T (3–4 vs. 1–2)	3.1	2.2–4.3	2·10^−11^			
N+	3.4	1.7–7.0	6·10^−4^			
M+	4.4	3.2–6.2	3·10^−18^	3.1	1.8–5.4	5·10^−5^
High stage (III–IV vs. I–II)	3.9	2.8–5.6	10^−14^	2.1	1.2–3.8	0.009
Low CUL5 expression	1.7	1.2–2.4	0.003	1.7	1.0–2.7	0.03
*CUL5* chromosomal deletion	2.0	1.3–3.2	0.002			

*HR* hazard ratio, *CI* confidence interval

In summary, low CUL5 gene expression is an independent negative prognostic factor in ccRCC.

## Discussion

*CUL5* was originally cloned as vasopressin-activated calcium-mobilizing (VACM-1)-encoding gene^29,30^. CUL5 is the least conserved of the cullin family members^31,32^ and it has gained attention through its role in the CRL-mediated degradation of APOBEC3G by human immunodeficiency virus Vif to thwart host cell antiviral defense mechanisms^33^. In cancer, downregulation of CUL5 has been found in a number of entities including breast cancer^30,34,35^, endometrial cancer^36^, cervical cancer^37^ and B-cell chronic lymphocytic leukemia^35^.

CUL5 is expressed in normal renal collecting tubule cells^38^ and the genomic locus of the *CUL5* gene, chromosome 11q22-23, has recently been implicated as a risk locus for RCC in a genome-wide association study^39^.

In the present report, we show that a reduced CUL5 gene expression or *CUL5* deletion is associated with significantly impaired overall survival in ccRCC patients and with more rapid tumor progression, respectively. Remarkably, low CUL5 expression was an independent prognostic factor in ccRCC, a tumor entity for which patient risk stratifiers are urgently needed.

Mechanistically, we show that loss of CUL5 can rapidly disrupt mitotic fidelity and induce structural chromosomal damage, very likely with an attenuation of DNA DSB repair as a strong contributing factor. In this regard, CUL5 loss may contribute to the extensive intratumoral heterogeneity that characterizes ccRCC and that is driven by genomic instability^8,9,40^. Chromosome 11q loss has not been found to represent a hotspot for somatic copy number alterations in the TRACERx cohort^3^ and our findings showing a *CUL5* deletion in approximately 10% of ccRCC patients are in line with this finding. Nevertheless, if such a *CUL5* loss is present, it confers a more rapid progression towards a lethal disease outcome (Fig. 4b).

CUL5 has previously been implicated to function as a tumor suppressor by regulating cellular proliferation^38^. CUL5 has been shown to be expressed in non-proliferating endothelial cells and downregulated during angiogenesis^41^. It is hence possible that downregulation or loss of CUL5 in ccRCC may further fuel neo-angiogenesis, which plays a central role in ccRCC driven by VHL loss, thus promoting tumor progression.

CUL5 has been shown to restrict Src activity^42^. Src is a potent inducer of tumorigenesis and has been implicated in both the regulation of cell division and DNA damage repair^43,44^. Whether Src or a different substrate of CUL5-based E3 ligase activity in fact mediates the observed cellular effects of a CUL5 loss on genome stability is the subject of future experiments.

Taken together, our results demonstrate that CUL5 is a novel candidate tumor suppressor in ccRCC that is involved in the maintenance of genome stability and has independent prognostic value in ccRCC patients.

## Materials and methods

### Cell culture and transfection

Human U-2 OS and HEK293 cells were obtained from ATCC and maintained in Dulbecco’s modified Eagle's medium (Cambrex, Walkersville, MD) supplemented with 10% fetal bovine serum (PAA, Pasching, Germany), 50 units/ml penicillin and 50 mg/ml streptomycin (PAA, Pasching, Germany). BJ/TERT fibroblasts were kindly provided by Ole Gjoerup (Dana-Farber Cancer Institute, Boston, MA, USA) and were maintained as reported previously^21^. U-2 OS and BJ/TERT cells were engineered to stably express a centrin-GFP-encoding construct (kindly provided by Michel Bornens, Institut Curie, Paris, France^45^). ACHN cells were obtained from Cell Line Services (Eppelheim, Germany) and maintained in Eagle's minimal essential medium supplemented with 10% fetal bovine serum (PAA, Pasching, Germany), 50 units/ml penicillin and 50 mg/ml streptomycin (PAA, Pasching, Germany). Normal human renal epithelial cells were obtained from Lonza (Basel, Switzerland) and maintained in Clonetics™ REGM™ Renal Epithelial Cell Growth Medium (Lonza, Basel, Switzerland). For transient transfections of U-2 OS (48 h), DN-CUL5 (provided by Wade Harper through Addgene) or empty vector controls were used and transfected by lipofection (Fugene 6; Roche). A vector encoding red fluorescent protein targeted to mitochondria (dsRed; BD Biosciences Clontech, Palo Alto, CA, USA) was used as transfection control. For transient transfection of hRECs (48 h), centrin-GFP was used and transfected using the Neon® Transfection System for Electroporation according to the manufacturer’s protocol (Invitrogen, Carlsbad, CA, USA).

### Immunofluorescence microscopy

Cells grown on 10 mm coverslips were permeabilized with 1% Triton-X-100 for 15 min, washed in phosphate-buffered saline (PBS) and then fixed in 4% paraformaldehyde/PBS followed by blocking in 10% normal donkey serum (Jackson Immunoresearch, West Grove, PA, USA). Cells were incubated with primary antibody overnight followed by incubation with a Rhodamine Red- or Coumarin (AMCA)-conjugated secondary antibody (Jackson Immunoresearch, UK) for 2 h and mounted with 4′,6-diamidino-2-phenylindole (DAPI). Cells were analyzed using an Olympus AX70 epifluorescence microscope equipped with a SpotRT digital camera. Antibodies used were mouse anti-BRCA1, rabbit anti-CUL5 and rabbit anti-53BP1 obtained from Santa Cruz Biotechnology (Santa Cruz, CA, USA). An anti-γH2AX antibody was obtained from Millipore. Mouse anti-Cep170 was a kind gift from Erich A. Nigg (Biozentrum, University of Basel, Switzerland)^24^.

### siRNA and shRNA

Synthetic RNA duplexes to reduce CUL5 protein expression were used (Flexitube, Qiagen, Valencia, CA, USA; Hs-CUL5_1 sense strand 5’-GGUUUGAAUCAGUCACCUATT-3’, antisense strand 5’-UAGGUGACUGAUUCAAACCTG-3’) according to the manufacturer’s protocol. For the cullin siRNA mini-screen, siRNAs were obtained from Qiagen (Flexitube; target sequences available upon request). shRNA vectors for CUL5 (TR313638) were obtained from OriGene Technologies, Inc. (Rockville, MD, USA).

### Immunoblot analysis

Cell lysates were prepared using an NP-40-based lysis buffer (1% NP-40, 50 mM Tris-HCl, pH 8.0, 100 mM sodium fluoride, 30 mM sodium pyrophosphate, 2 mM sodium molybdate, 5 mM EDTA, 2 mM sodium orthovanadate in dH_2_O) containing protease inhibitors (10 μg/ml aprotinin, 10 μg/ml leupeptin, 1 μM phenylmethylsulfonyl fluoride, 2 M vanadate). After 1 h rotation at 4 °C, lysates were cleared by centrifugation for 30 min at 13,000 rpm at 4 °C. Protein concentrations were determined using the Bradford assay (Bio-Rad Laboratories, Hercules, CA, USA). Then, 30 μg of protein was loaded on a 4–12% Bis-Tris or 3-8% Tris-Acetate gel (Invitrogen, Carlsbad, CA, USA) and blotted onto a nitrocellulose membrane.

Antibodies directed against CUL5, p21^Cip1^ (F-5), p53 (DO-1) and PCNA (PC10) were obtained from Santa Cruz Biotechnology (Santa Cruz, CA, USA). An antibody directed against actin (AC-42) was purchased from Sigma (St. Louis, MO, USA).

### Quantitative real-time PCR

For quantitative real-time polymerase chain reaction (qPCR), RNA was extracted using the RNeasy Mini Kit (Qiagen, Valencia, CA, USA) according to the manufacturer’s protocol. Extracted RNA was first treated with DNase I enzyme (Fermentas, St. Leon-Rot, Germany) according to the manufacturer’s protocol to remove any contaminating traces of genomic DNA. Complementary DNA (cDNA) was then transcribed by RT-PCR using random primers and the Maxima First Strand cDNA Synthesis Kit (Fermentas, St. Leon-Rot, Germany) according to the manufacturer’s protocol. qPCR was then performed using specific primers to *CUL5* (forward: 5’-GAACACAAGCACCCTCGTATT-3’, reverse: 5’-TCAACGGAGTTACATTCTCGTCT-3’; IDT, Leuven, Belgium) and actin (forward: 5’-CCAAGGCCAACCGCGAGAAGATGAC-3’, reverse: 5’-AGGGTACATGGTGGTGCCGCCAGAC-3’). *CUL5* cDNA was amplified and measured using the SsoFast EvaGreen Kit (Bio-Rad, Hercules, CA, USA) according to the manufacturer’s protocol. Cycling conditions were 95 °C (30 s, activation), 95 °C (5 s, denaturation) and 60 °C (10 s, annealing/extension) for 40 cycles for CUL5 amplification on a Bio-Rad CFX96 Real-Time System run on a C1000 Thermal Cycler platform (Bio-Rad, Hercules, CA, USA). Actin cDNA served as reference for relative quantification.

### Immunohistochemistry

Briefly, sections from a commercially available tissue microarray (US Biomax) were deparaffinized in xylene, rehydrated in a graded ethanol series and boiled in a microwave oven for 30 min in citrate buffer (pH 6.0) followed by blocking and incubation with a primary anti-CUL5 antibody (Sigma, at a 1:50 dilution). Immunodetection of the primary antibody was performed using the HistoStain PLUS kit (Invitrogen, Germany) according to the manufacturer’s recommendations.

### Cell cycle analysis

For cell cycle analysis, U-2 OS/centrin-GFP cells were transfected with siRNA duplexes against CUL5 mRNA (see above) and assayed for cell cycle distribution after propidium iodide staining at 72 h post transfection. Briefly, cells were trypsinized and pelleted by centrifugation. The cell pellet was then washed two times with PBS prepared without calcium or magnesium. After washing in PBS, the cells were resuspended in 70% ethanol and fixed overnight at 4 °C. The next day, the cells were again pelleted and washed two times with PBS/1% bovine serum albumin (BSA) to prevent clumping. After the final centrifugation, the cell pellet was suspended in 800 μl PBS/1% BSA. The cells were then mixed with 100 μl propidium iodide (0.5 mg/ml) and 100 μl boiled RNase A (10 mg/ml) and incubated at 37 °C for 30 min. The propidium iodide-stained cells were protected from light and DNA content was analyzed on a FACSCalibur flow cytometer.

### Comet assay

For the analysis of DNA damage in CUL5-deficient cells, U-2 OS/centrin-GFP cells were transfected with siRNA duplexes against CUL5 mRNA (see above for protocol) for 72 h and efficiency of DNA repair was analyzed with or without 10 Gy IR using the alkaline Comet assay (Trevigen, Gaithersburg, MD, USA). Briefly, cells were trypsinized and pelleted by centrifugation. The cell pellet was then washed two times with PBS prepared without calcium or magnesium and cells were resuspended at a concentration of 1 × 10^5^ cells/ml in PBS. Cells were then mixed with LMAgarose at a 1:10 ratio and 50 μl of this solution was pipetted onto Comet assay slides. The slides were placed at 4 °C in the dark for 10 min until the cell/agarose solution hardened. The slides were then immersed in cold lysis solution provided with the kit for 1 h at 4 °C in the dark. Slides were subsequently immersed in alkaline unwinding buffer (30 mM NaOH, 1 mM EDTA) for 1 h at 4 °C in the dark. Next, alkaline electrophoresis was performed using alkaline electrophoresis solution (300 mM NaOH, 1 mM EDTA) at 300 mA for 30 min. Slides were washed two times for 5 min each in dH_2_O and once for 5 min in 70% ethanol before being stained with SYBR Gold and analyzed on an Olympus AX70 epifluorescence microscope equipped with a SpotRT digital camera.

### TCGA data and statistical analysis

RNA-Sequencing (RNA-Seq) and clinical data of clear cell renal cell carcinoma (KIRC) were downloaded from TCGA data portal (https://tcga-data.nci.nih.gov/tcga) on 10 December 2014. Correlations between genomic and clinical data were performed as previously described^46^. Briefly, the RNA-Seq Expectation-Maximization (RSEM) normalization method was used for the gene expression analysis. Gene expression was stratified based on quartiles (1st quartile = low expression, 2nd and 3rd quartiles = intermediate expression, 4th quartile = high expression). To compute overall survival, the patient date of death of any cause or the last date the patient was known to be alive was considered. IBM SPSS Statistics v25 was used to calculate the Kaplan–Meier survival curves, log-rank tests and univariate and multivariate Cox regression models. For all other analyses, Student’s *t*-test for independent samples (two-tailed) was used wherever applicable. Three independent replicates were performed for all experiments or indicated otherwise.

## Supplementary information

Supplemental Material
