# Supplementary material for: Cullin 5 is a novel candidate tumor suppressor in renal cell carcinoma involved in the maintenance of genome stability
Source: Oncogenesis. 2019 Jan 9;8(1):4. doi: 10.1038/s41389-018-0110-2 (PMC6328621; doi:10.1038/s41389-018-0110-2)
Supplement: Supplementary file 1 — Supplemental Material [file 41389_2018_110_MOESM1_ESM.docx]

**SUPPLEMENTAL MATERIAL**

**
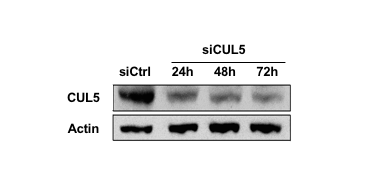
**

**Suppl. Figure 1. Downregulation of CUL5 by siRNA.**

Immunoblot analysis of U-2 OS-centrin/GFP cells transfected for 72 h with either control or siRNA duplexes targeting CUL5. Immunoblot for actin demonstrates protein loading.

**
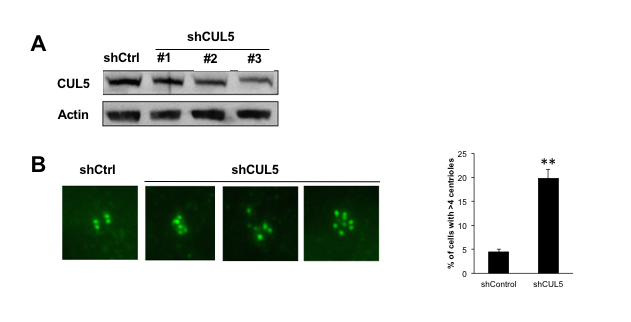
**

**Suppl. Figure 2. Dowregulation of CUL5 by shRNA in BJ/Tert fibroblasts induces centriole overduplication.**

(A) Immunoblot analysis of BJ/Tert fibroblasts stably expressing centrin-GFP for CUL5 expression after transfection with three shRNA plasmids. An immunoblot for actin is shown to demonstrate protein loading.

(B) Fluorescence microscopic analysis (left panels) and quantification (right panel) of BJ/Tert fibroblasts stably expressing centrin-GFP for centriole overduplication induced by shRNA-mediated CUL5 knock-down (96 h). Bars represent mean and standard error of two independent experiments.
